# Cell cycle-dependent and -independent telomere shortening accompanies murine brain aging

**DOI:** 10.18632/aging.101655

**Published:** 2018-11-20

**Authors:** Quratul Ain, Christian Schmeer, Diane Penndorf, Mike Fischer, Tzvetanka Bondeva, Martin Förster, Ronny Haenold, Otto W Witte, Alexandra Kretz

**Affiliations:** 1 Hans Berger Department of Neurology, Jena University Hospital, 07747 Jena, Thuringia, Germany; 2 Department of Internal Medicine II, Jena University Hospital, 07747 Jena, Thuringia, Germany; 3 Department of Internal Medicine III, Jena University Hospital, 07747 Jena, Thuringia, Germany; 4 Department of Internal Medicine I, Jena University Hospital, 07747 Jena, Thuringia, Germany; 5 Leibniz Institute on Aging – Fritz Lipmann Institute, 07745 Jena, Thuringia, Germany; 6 Matthias Schleiden Institute for Genetics, Bioinformatics and Molecular Botany, Friedrich Schiller University Jena, 07743 Jena, Thuringia, Germany; * Equal contribution

**Keywords:** cell cycle, cellular senescence, brain aging, RelA subunit of nuclear factor kappa B, telomere length, telomerase reverse transcriptase

## Abstract

Replication-based telomere shortening during lifetime is species- and tissue-specific, however, its impact on healthy aging is unclear. In particular, the contribution of telomere truncation to the aging process of the CNS, where replicative senescence alone fails to explain organ aging due to low to absent mitotic activity of intrinsic populations, is undefined. Here, we assessed changes in relative telomere length in non-replicative and replicative neural brain populations and telomerase activity as a function of aging in C57BL/6 mice. Telomeres in neural cells and sub-selected neurons shortened with aging in a cell cycle-dependent and -independent manner, with preponderance in replicative moieties, implying that proliferation accelerates, but is not prerequisite for telomere shortening. Consistent with this telomere erosion, telomerase activity and nuclear TERT protein were not induced with aging. Knockdown of the *Rela* subunit of NF-κB, which controls both telomerase enzyme and subcellular TERT protein allocation, did also not influence telomerase activity or telomere length, in spite of its naive up-regulation selectively under aging conditions. We conclude that telomere instability is intrinsic to physiological brain aging beyond cell replication, and appears to occur independently of a functional interplay with NF-κB, but rather as a failure to induce or relocate telomerase.

## Introduction

Telomeres are protein-DNA structures, with the DNA component consisting of tandem repeats of (TTAGGG)_n_ hexanucleotide sequences that protect chromosomal ends from attrition, improper fusion and induction of DNA damage responses (DDR). Telomere erosion after repeated cell cycles induces cellular senescence in replicative tissues and is considered to be one of the hallmarks of organ aging. Consistently, telomeres are essential in maintaining the replicative capacity of cultured cells [1]. However, their relationship with senescence and lifespan *in vivo* remains poorly understood [2]. Telomere repeats are heterogeneous in inter-individual length both in rodents and humans, and their absolute dimension is weakly correlated with cell turnover rates in defined tissues and ultimate lifespan of an organism [3,4]. Likewise, in humans telomeric repeats are 5-15 kilo base pairs (kbp) long [5], whereas in short-lived mice they can be highly variable, with 5-20 kbp for feral *Mus spretus* [6] and 30-150 kbp for the laboratory mouse *Mus musculus* [6,7]. Thus, due to little knowledge about organ-specific telomere dynamics over lifetime, the correlation with age-related loss of tissue functions and vitality is still not understood. In particular, the role of telomere length alterations and their participation in the healthy aging process of the central nervous system (CNS) and in neurosenescence at the cellular level are incompletely understood [4]. Furthermore, age-related changes specifically in neurons are still understudied.

Cell cycle activity as a driving force for telomere attrition has traditionally been assumed to be absent in neurons once they achieved their terminal differentiation. This view has been challenged by the discovery of DNA content variations apparently indicating a cell cycle re-induction in about 10-20% of post-mitotic neurons, as described for the cortex of healthy aging brains and in Alzheimer’s disease [8,9]. In this context, an open question remains whether a putative telomere shortening in neural cell populations may eventuate by unscheduled abortive cell division cycles, or occur even independently of any cell cycle activity.

Telomere length is maintained by the enzyme telomerase, which adds (TTAGGG)_n_ repeats to telomere endings. In adult somatic tissues including the CNS, telomerase shows very low activity and transcript levels [10,11], which are inconsistent regarding their correlation with protein levels, e.g., in murine cortex [12]. Moreover, TERT protein displays a maturation-dependent allocation to different subcellular compartments, thereby exhibiting a shift from nuclear preponderance in embryonic to cytoplasmic prevalence in adult cortex [12]. Differences in spatial TERT distribution and localization, e.g., to mitochondrial versus nuclear structural components also argue for telomere-independent functions, as shown for cell viability and tissue homeostasis [13], and in relation to DNA structure stabilization and contribution to DDR in several tissues including the CNS [14].

This study aimed to evaluate the impact of physiological aging on telomere length alterations and telomerase activity in brain tissue, as exemplified for murine neocortex, with particular emphasis on neuronal cell moieties. Using Flow-FISH techniques, changes in the relative telomere length (RTL) were first dissected for replicative and non-replicative neural populations as a function of aging in a C57BL/6 wild type mouse colony aged up to 25-27 months. Age-dependent alterations in cortical RTL were confirmed and further specified for neurons by qPCR-based telomere length analysis, and correlated with telomerase activity and telomerase inductive NF-κB *Rela* transcript levels, the second being a master regulator of age-related genetic reprogramming.

## RESULTS

### Relative telomere length of cortical neural cells in G_0_/G_1_ phase is reduced in the aged brain

**Figure 1:**
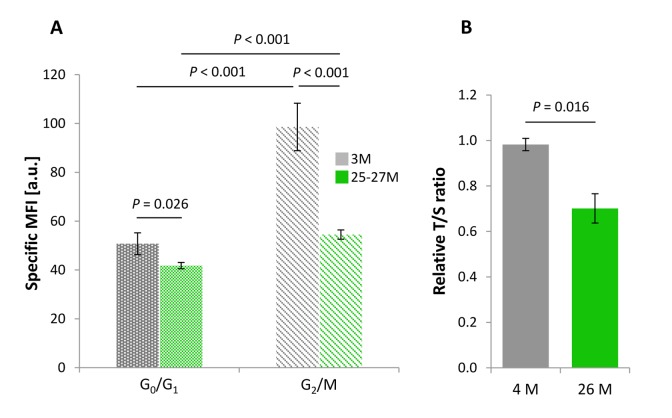
**Age-dependent changes in the RTL of cortical neural cell isolates.** (**A**) RTL assessed by Flow-FISH as a function of cell cycle activity. PNA-FITC-related mean fluorescence intensity corrected against background signal (specific MFI) was taken as an indirect parameter for RTL in cells at G_0_/G_1_ and G_2_/M phases of the cell cycle. For both cell populations, a significant age-related reduction in RTL was observed. Bars represent means ± SEM (*n* = 4 - 8). *P*-values were assessed with Two-way ANOVA. (**B**) Cell cycle-independent measurement of RTL assessed by qPCR and determined in terms of a relative T/S-ratio that was referenced against a 4-month-old control group. RTL declined significantly in cortical neural cells of aged as compared to young samples, thus confirming the results obtained in (**A**). Bars represent means ± SEM (*n* = 3 - 4). *P*-values were calculated using the Student’s *t*-test.

RTL of cortical neural cells residing in G_0_/G_1_ phase of the cell cycle was significantly reduced in mice aged up to 25-27 months (*n* = 8) compared with young gender-matched counterparts at an age of 3 months (*n* = 4). Accordingly, the absolute PNA-FITC-specific mean fluorescence intensity (MFI) corrected against background signal (specific MFI) for aged and young neural cells accounted for 41.81 a.u. and 50.76 a.u., respectively (Figure 1A, dotted bars). This corresponded to a decline in specific MFI by 17.64% (*P* = 0.026). Such a reduction in specific MFI reflects a significant age-dependent loss in mean telomere repeat length.

Autofluorescence increased in aged relative to young samples both with respect to the PI- and the FITC-detecting signals. However, when exemplified for 4.5 months versus 22 months old animals a change from 0.457 ± 0.067% overlap with the specific channel in the young specimens to 1.382 ± 0.423% overlap in the samples from old isolates for the telomere-related PNA-FITC signal reflects that sensitivity was preserved for >98% of the cells.

### Relative telomere length of cortical neural cells in G_2_/M phase is strongly reduced by aging

RTL was further examined in neural cortical cells that exited G_0_/G_1_ phase of the cell cycle and adopted features of G_2_/M phase, as identified by FACS gating strategies on the basis of their increased DNA content. RTL of G_2_/M phase cells isolated from 25-27 months aged rodents was drastically reduced by 44.72% (*n* = 8) compared with neural populations derived from young animals *(n* = 4), as reflected by an absolute decline in specific MFI from 98.58 a.u. to 54.49 a.u. with age (*P* < 0.001; Figure 1A, striped bars).

### RTL measures by qPCR correspond to Flow-FISH-based RTL results

Since Flow-FISH-based protocols for telomere length evaluations are primarily established for blood cells, we previously assessed nuclear localization of PNA-FITC-specific fluorescence also in nuclei of murine cortical neurons and the lymphoma cell line L5178-R. As shown in Figure S1, after hybridization the PNA-FITC-specific signal was co-localized with neuronal and nuclear markers in cortical neurons (Figure S1A), displaying a signal pattern similar to that in nuclei derived from the lymphoma cell line, both in interphase and metaphase (Figure S1B and C). For validation of absolute telomere length attained by our protocol, the human tetraploid T-cell leukemia 1301 cell line with extremely long telomeres was employed as an internal reference. Underlying a telomere length of ~ 90 kbp for this cell line [15], the results obtained for our murine neural isolates indicate an appropriate telomere repeat content of ~ 84 kbp and ~ 98 kbp in G_0_/G_1_ and G_2_/M phases, respectively (Figure S2), which is in the range reported for *Mus musculus* [6,7].

In order to further validate our protocol and results on cortical neural RTL achieved by Flow-FISH, we applied a standard quantitative real time PCR approach, according to a protocol by O’Callaghan and Fenech [16]. Telomere repeat copies amplified from each sample were referenced against a single copy gene amplification product and expressed as T/S-ratio. One limitation of the qPCR approach is that it does not support a cell cycle phase-specific RTL assessment. As illustrated in Figure 1B, analysis of cortical cell isolates including all active cell cycle phases showed a significant age-dependent decline in the RTL by 28.61% when assessed as T/S-ratio (*P* = 0.016; Figure 1B) and thus confirmed the Flow-FISH-based results.

### Age-related telomere shortening of cortical neural cells is not caused by changes in cell composition

**Figure 2:**
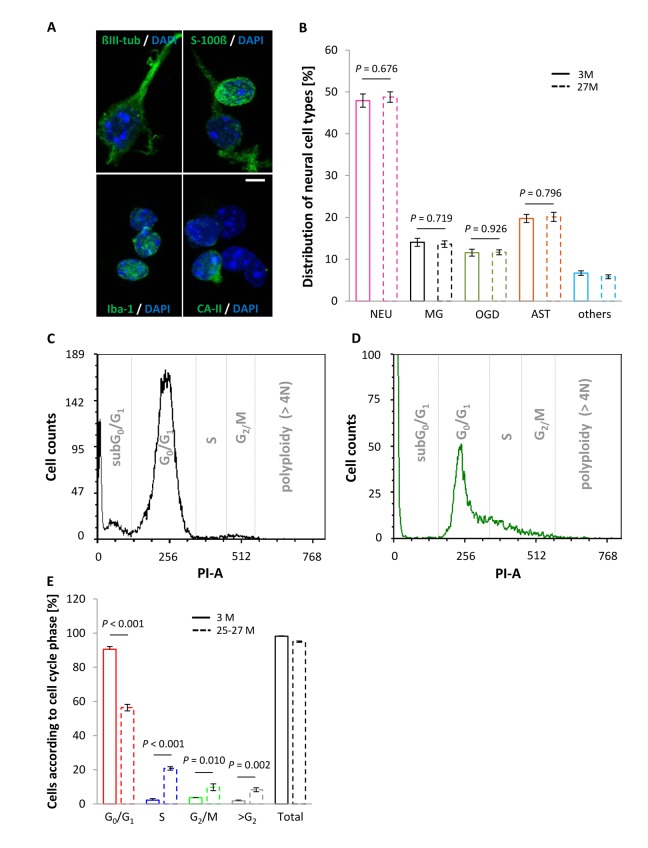
**Neural cell type proportions and DNA content in the vital fraction of cortical cell isolates as a function of age.** (**A**) Cellular entities were identified by applying cell type-specific markers, using ßIII-tubulin, S100-ß, Iba-1 and CA-II for neurons, astrocytes, microglia and oligodendrocytes, respectively, with cell nuclei being discriminated by DAPI. Scale bar, 5 µm. (**B**) Cell type-specific proportions did not vary between young and aged mice. NEU, neurons; MG, microglia; OGD, oligodendrocytes; AST, astrocytes. Bars represent means ± SEM (*n* = 500 cells out of 3 animals per condition). (**C** and **D**) Representative DNA histograms illustrating an age-associated shift in cell cycle activity, as assessed by PI staining of DNA from murine cortical neural cell isolates at an age of 3 months (**C**) and 25-27 months (**D**). (**E**) Neocortical cell constituents derived from aged animals showed a shift towards replicative cell cycle phases, marked by a significant increase in the proportion of cells in S, G_2_/M and >G_2_ phase, whereas the percentage of cells in G_0_/G_1_ phase declined significantly at higher age. Bars represent means ± SEM (*n* = 4 - 8). For (**B** and **E**) *P*-values were calculated using the Student’s *t*-test.

To confirm that the observed age-associated changes in RTL of cortical neural cells are not influenced by variations in the composition of neural cell types with either moderate or absent cell cycle activity, the proportions of different neural identities were assessed in cortical cell isolates at different ages. Neurons accounted for 47.93 ± 1.59% and 48.78 ± 1.25% in lysates from young mature and aged animals, respectively (*P* = 0.676; Figure 2B) indicating that their relative abundance was not influenced by aging. The vast majority of the vital cells, of which approximately every second exhibited a neuronal identity (Figure 2A and B), were found in G_0_/G_1_ phase at either age category (Figure 2E). Moreover, together with the neuronal moiety, non-neuronal glia populations comprising astrocytes, oligodendrocytes and microglia, amounted up to 93.31 ± 0.54% (3 months) and 94.19% ± 0.46% (27 months) of the total cortical cell isolate, and thus did also not shift with age (Figure 2A and B). The remaining 5-7% of uncharacterized cells is most likely of endothelial origin. In summary, age-related differences in RTL were not affected by cell composition.

Notably, an age-associated increase in the number of neural cells entering the cell cycle and displaying a DNA content characteristic of an S phase state or G_2_/M phase was detected, as delineated in the DNA histograms given in Figure 2C and D and quantified in Figure 2E. Moreover, although the proportion of aneuploid cells, discerned by a DNA content of more than 4N, was negligible in young animals (1.76 ± 0.48%, *n* = 4) it significantly augmented in aged animals up to 8.28 ± 1.15% (*n* = 8), as indicated by the >G_2_/M bar in Figure 2E (*P* = 0.002). This suggests that polyploidy/aneuploidy is a rare event in the young and healthy brain but may increase in the aged CNS.

### Higher mean fluorescence intensity in G_2_/M phase cell populations is proportionate to the increased telomeric DNA content

**Figure 3:**
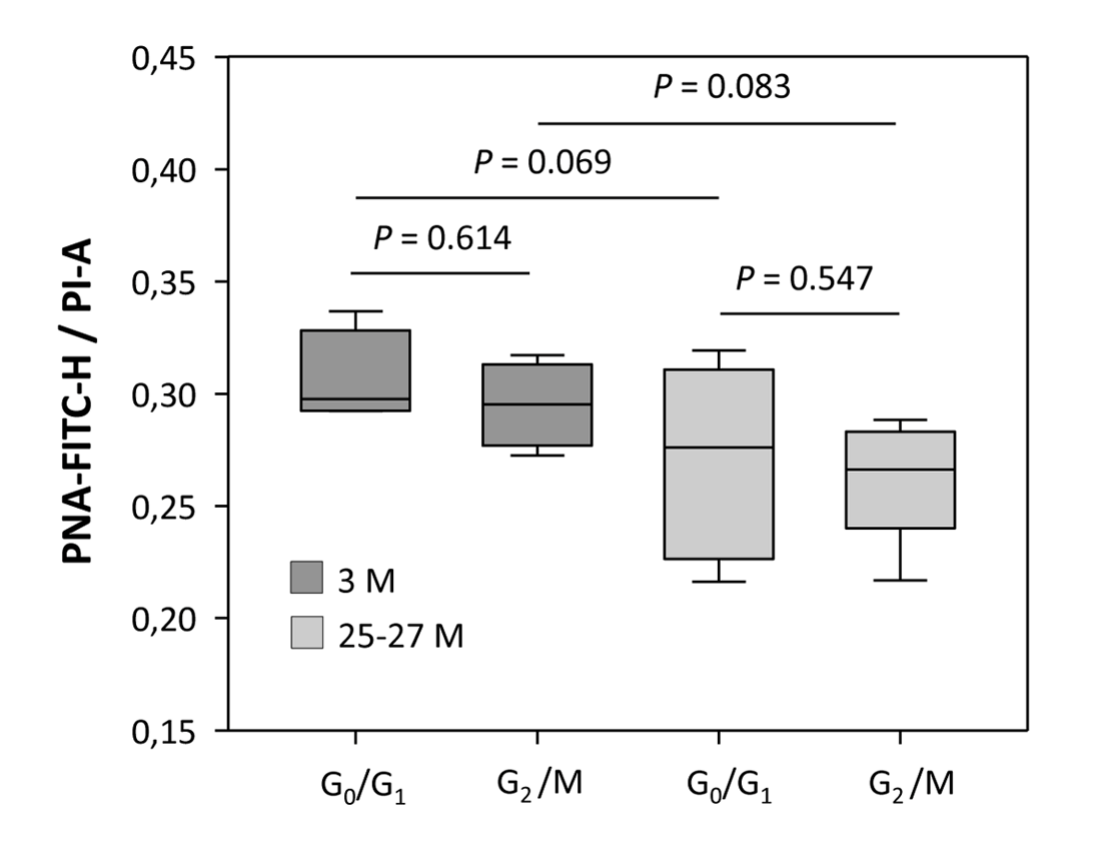
**Relationship between telomere-related DNA and the total DNA content at different cell cycle phases, assessed for young and aged cortical neural cell isolates**. Telomere-related DNA (PNA-Fluorescein Isothiocyanate height [PNA-FITC-H]-specific MFI) was normalized against the total DNA content (propidium iodide area [PI-A]-specific MFI). The ratio of PNA-FITC-related MFI corrected against the DNA content of G_0_/G_1_ and G_2_/M phase displayed stable values for both young and aged groups. Bars represent means ± SEM (*n* = 4 - 8). *P*-values were calculated using the Student’s *t*-test.

Cell fractions attributable to G_2_/M phase of the cell cycle from both young and aged animals displayed an overall higher specific MFI level than cell populations in G_0_/G_1_ phase (*P* < 0.001 for both comparisons; Figure 1A), probably due to the increased DNA content intrinsic to nuclei beyond S phase and hence an augmented telomere-specific DNA content per cell. As delineated in Figure 1A, the corrected telomere-related MFI values of G_2_/M phase populations were highest in young animals, as indicated by a 94.2% increase relative to G_0_/G_1_ populations (*P* < 0.001). Aging attenuated this increase relative to G_0_/G_1_ phase levels to 30.34% (*P* < 0.001, Figure 1A). To ascertain that this increase in specific MFI of G_2_/M cells is due to doubled DNA content rather than to intrinsically longer telomere tracts, absolute values for PNA-FITC-related MFI were normalized against the sample-specific total DNA content (Propidium iodide area, [PI-A]) for both the G_0_/G_1_ and G_2_/M phase cell fractions, and compared between young and advanced ages. The ratios obtained ranged between 0.306 ± 0.011 and 0.295 ± 0.009 in the young group (*P* = 0.614) and 0.270 ± 0.014 and 0.261 ± 0.009 in the aged group (*P* = 0.547) for G_0_/G_1_ and G_2_/M, respectively (Figure 3). The stable ratios for G_0_/G_1_ and G_2_/M cells, irrespective of age, exclude that the elevation in the PNA-FITC-related MFI of G_2_/M phase samples is due to an increase in telomere length, but argues for an origin from amplified telomere tract numbers subsequent to the S phase transition. It also supports that the age-associated decline in specific MFI of both cellular populations residing either in G_0_/G_1_ or G_2_/M phase is a consequence of actual telomere shortening rather than any artifact arising from a decreased DNA content. Accordingly, when the DNA content of cortical neural cells was referenced against the nuclear DNA amount of trout erythrocytes as a positive control, the ratios of 1.089 ± 0.0022 (*n* = 4) and 1.087 ± 0.0004 (*n* = 4) obtained for young and aged cell isolates, respectively (*P* = 0.352), as illustrated in Figure S3, were both in accordance with the ratio of 1.086 predicted from the animal genome sizes (http://www.genomesize.com). These measures thus prove that the MFI calculations are based on a standard diploid genome of murine origin.

### Cell cycle activity represented by G_2_/M phase cortical neural cells is a putative driving force for age-dependent telomere shortening

**Figure 4:**
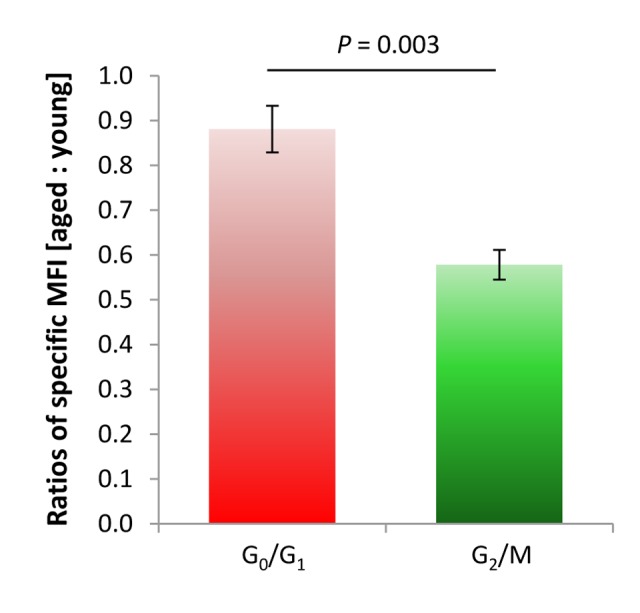
**Rate of age-dependent changes in RTL of cortical neural isolates from murine neocortex, for G_0_/G_1_ and G_2_/M phases of the cell cycle.** A lower specific MFI ratio between aged and young specimens for G_2_/M-phase cells as compared to a more stable specific MFI ratio for moieties arrested at G_0_/G_1_ phase reflected a substantial age-related decline in RTL in replicative cell entities in the murine cortex over lifetime. Bars represent means ± SEM (*n* = 4 - 8). *P*-values were calculated using Student’s *t*-test.

The age-dependent decline in RTL of G_0_/G_1_ phase populations was moderate as reflected by an aged:young RTL ratio close to 1.0, i.e. of 0.88 ± 0.05, whereas the age-related decrease in RTL was drastic for actively cycling G_2_/M phase cell populations, displaying a diminished ratio of 0.58 ± 0.03 (*P* = 0.003, Figure 4). Thus, though not exclusive for cells with replicative features, cell cycle activity represented by cell cycle phases beyond G_0_/G_1_ might constitute a driving force for age-dependent telomere shortening also in the CNS.

### Cortical neurons undergo telomere shortening with aging

**Figure 5:**
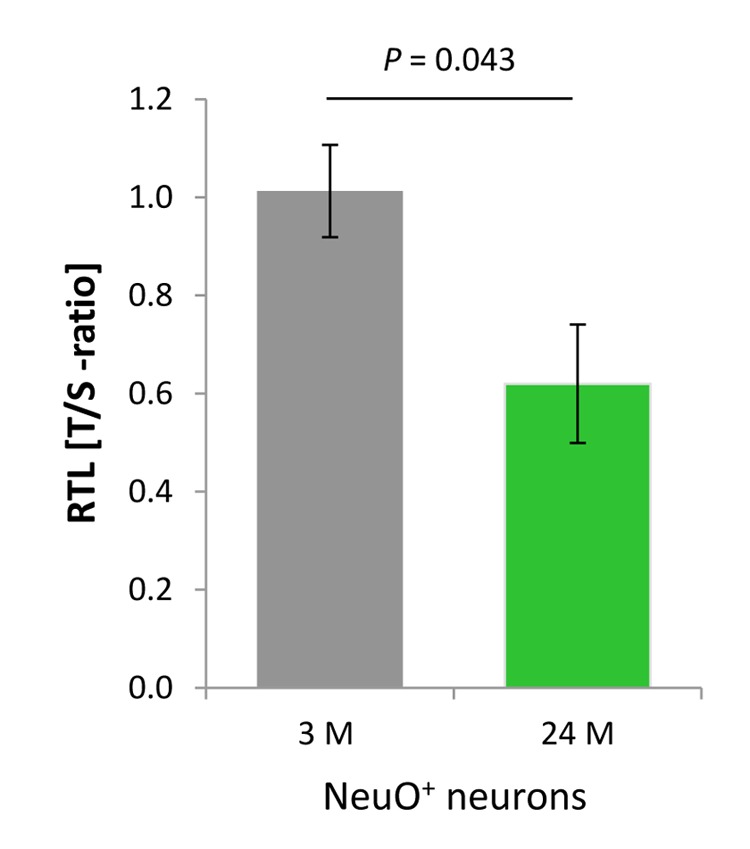
**Age-dependent telomere length dynamics in the neuronal fraction sorted from murine cortical isolates.** NeuO^+^ neurons showed a significant age-related decline in RTL as determined by qPCR in terms of telomere repeat (T) to single copy gene (S) ratio. Bars represent means ± SEM (*n* = 4). *P*-values were calculated using Student’s *t*-test.

As delineated in Figure 2B, approximately 50% of the cells in the cortical neural cell isolates possess neuronal identity. This raised the question whether neurons, despite being primarily postmitotic, are also involved in the age-associated decline in RTL defined for the aged cerebral cortex. In order to determine the contribution of neurons to this telomere erosion process, neuronal RTL was assessed using a qPCR-based strategy subsequent to FACS of cells positive for the neuron-specific NeuroFluor™ NeuO marker, as the NeuO approach was incompatible with the Flow-FISH technique used previously for RTL assessment. The T/S-ratio of cortical neurons, which relates the telomere repeat amplicons to the 36B4 single copy gene amplification product, declined with aging by 38.8% (*P* = 0.043, Figure 5) indicating that post-mitotic neurons in the aging murine cortex are subjected to substantial telomere shortening.

### Activity of telomerase enzyme is unchanged in the aging cortex

**Figure 6:**
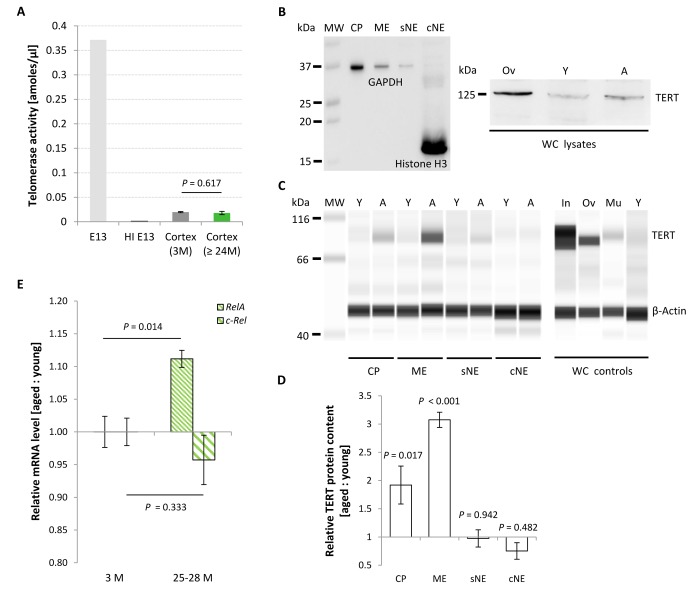
**Age-associated changes in TERT protein content, telomerase activity and relative *Rela* and *c-Rel* expression levels in the murine cortex.** (**A**) Telomerase activity assessed from cortical isolates as a function of age. Telomerase activity in the mature neocortex remained unchanged with aging, displaying activity levels approximately 20 times lower than in E13 brain. Bars of probe samples represent means ± SEM (*n* = 5). HI E13, heat inactivated E13 protein extract. (**B**-**D**) Relative TERT protein levels in different subcellular compartments of murine cortical tissue at young (Y) and aged (A) states. (**B**) Left blot: Conventional Western blot. Purity of the cellular subfractions is illustrated by the enriched levels of the nuclear and cytoplasmic marker proteins Histone H3 and GAPDH in the chromatin-bound nuclear (cNE) and cytoplasmic (CP) fractions, respectively. Right blot: TERT product at the appropriate molecular weight of ~ 125 kDa in whole cell (WC) lysates from ovary (Ov) and cortex of young (Y) and aged (A) mice. (**C**) Simple Western™ technique. Little or no TERT protein was expressed in the soluble nuclear (sNE) and chromatin-bound nuclear (cNE) fraction, respectively, at both age categories, whereas TERT protein was found at significantly higher levels in the cytoplasmic (CP) and membrane (ME) fractions at 27 months of age. Specificity of the TERT antibody was proven by the detection of different TERT protein levels in whole cell (WC) lysates prepared from small intestine (In), ovary (Ov), muscle (Mu) and cortex of young mice (Y). All cortical samples were assayed in the same run, as were all the control samples. (**D**) Quantification of the compartment-specific TERT protein levels for young and old cortices as illustrated in (**C**). Bars represent means ± SEM (*n* = 3), and were reproduced in two independent runs. (**E**) Age-associated changes in relative mRNA levels of the subunits *Rela* and *c-Rel* of canonical NF-κB*.* *Rela* transcription levels increased significantly at advanced ages, whereas levels of *c-Rel* remained unaltered. Ratios represent geometric means of *n* = 5 animals ± SEM. *P*-values were calculated using Student’s *t*-test (**A**) and Two-way ANOVA (**D** and **E**).

The catalytically active TERT and telomerase RNA component are the most important units of the telomerase holoenzyme. Telomerase activity was measured using a standard qPCR-based TRAP assay and referenced against the intrinsic activity of a HeLa cell-derived positive cell extract according to Klapper and colleagues [11], or expressed in absolute values. These were found to be low in mature cortex as compared to E13 brain (Figure 6A) and remained unchanged with chronological aging, as demonstrated by the comparably weak activity levels in 3 months young and 24-27 months aged murine neocortices (*P* = 0.617; Figure 6A). The observed reduction in RTL with aging might therefore be a result of the intrinsic inability of the murine brain to induce telomerase activity, which otherwise could compensate for telomere erosion.

### Subcellular content of TERT protein changes with aging

In the murine brain, telomerase enzyme activity shows only inconsistent correlation with TERT mRNA and protein levels [11,12], thus suggesting the involvement of TERT protein in non-canonical functions unrelated to telomere replenishment [14]. Moreover, TERT protein persists in adult neurons but not astrocytes [17], and its allocation to subcellular compartments varies with cellular conditions [13,14]. In order to reveal putative age-associated changes in TERT protein functions, we further assessed its content in different subcellular fractions isolated from murine neocortex, including neurons. Purity grade of the subcellular fractions was assessed by western blot, illustrating Histone H3 presence selectively in the chromatin-bound nuclear fraction (cNE) and a strong enrichment of the marker protein GAPDH in the cytoplasmic fraction (CP) (Figure 6B, left side). As a multifunctional protein, GAPDH is well established to exist not only in the cytoplasm but in diverse subcellular locations including membrane, cytoskeletal and nuclear compartments [18]. The soluble (sNE) and chromatin-bound (cNE) nuclear fractions were chosen to assess canonical chromatin-associated TERT functions, whereas cytoplasmic (CP) TERT would possibly reflect alternative cellular assignments and functions within organelles such as mitochondria [14]. A membrane fraction (ME) was included to assess TERT protein levels in the cellular membranes, comprising organelle-derived ones. TERT protein both in the sNE and cNE fractions was low to absent, and did not change between young and aged cellular states (0.97 ± 0.15-fold for sNE; *P* = 0.942; 0.75 ± 0.15-fold for cNE; *P* = 0.482; Figure 6C and D). In contrast, TERT protein levels in CP and ME sub-fractions significantly increased with aging, thereby reaching fold changes of 1.92 ± 0.34 (*P* = 0.017) and 3.08 ± 0.13 (*P* < 0.001) above expression levels in young controls, respectively (Figure 6C and D). Levels of the reference protein ß-Actin were not altered with aging. Specificity of the polyclonal anti-TERT antibody used in this study was verified by probing TERT control tissue lysates in parallel with the sub-fractionated cortical samples, demonstrating a strong TERT product in whole cell (WC) lysates of replication-competent small intestine and ovaries, whereas only a weak product was obtained from post-replicative skeletal muscle, and a very faint signal was detected from young cortical whole cell lysates (Figure 6C). The discrepancy between the predicted size of 123 kDa for murine TERT and the displayed molecular weight of approximately 93 kDa is most likely due to the intrinsic technical properties of the electrophoresis-based Simple Western™ system, which is based on a non-polyacrylamide matrix known to have protein motility properties and protein-matrix interaction features different from a standard polyacrylamide separation matrix. In order to prove this notion, we probed the samples with the same antibody in a conventional western blot. This approach resulted in a specific product of ~ 125 kDa size (Figure 6B, right side). Thus, the antibody used proved specificity and was appropriate to assess varying levels of TERT protein content in different murine tissues.

### Levels of the Nuclear Factor kappa B subunit *Rela* increase in the aged neocortex

NF-κB is a master regulator of age-related genetic reprogramming [19], and its up-regulation is a genetic signature of aging-associated inflammation, including neuro-inflammation [20]. Moreover, NF-κB RelA activity was shown to be linked to telomerase via a feed-forward loop, in which RelA and either telomerase holoenzyme or TERT protein can reciprocally activate each other [21,22].

**Figure 7:**
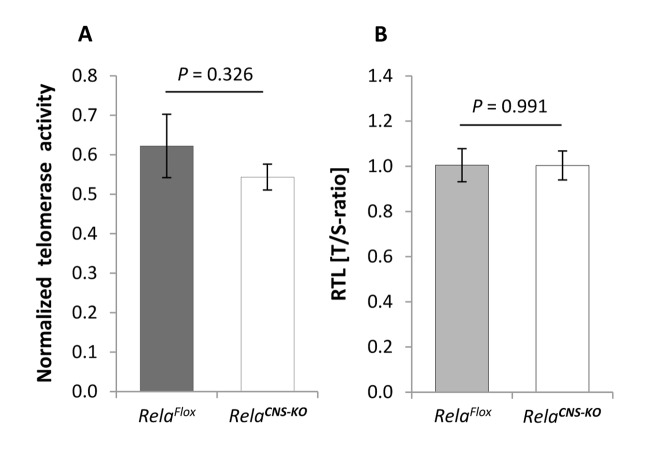
**Telomerase activity and telomere length in the brain of aged *Rela^CNS-KO^* mice.** (**A**) Telomerase activity, normalized against a positive HeLA cell extract, in the cortex of 18-23 months old *Rela****^CNS-KO^*** mice compared to age-matched *Rela^Flox^* control mice (*n* = 4 - 6). (**B**) RTL in cortical neural cells isolated from 19-20 months old *Rela****^CNS-KO^*** as assessed by qPCR in terms of T/S-ratios and normalized against aged-matched *Rela^Flox^* control animals (*n* = 3 - 4). Bars represent means ± S.E.M. *P* values were calculated by Student’s *t*-test.

To test a possible link between telomerase activity and NF-κB RelA in the CNS *in vivo*, in a first step we determined NF-κB *Rela* gene expression in the neocortex of young and aged mice. As expected, levels of *Rela* mRNA were significantly up-regulated in animals with advanced ages *(n* = 5) compared with young counterparts (Figure 6E; *P* = 0.014; *n* = 5). In contrast, levels of *c-Rel*, a heterodimer partner of RelA also involved in classical NF-κB cascade activation*,* though without hitherto evidence for a direct interaction with TERT were not substantially changed (*P* = 0.333). To further explore a putative mechanistic interaction between TERT and NF-κB RelA, telomerase activity was measured in the cortex of aged *Rela****^CNS-KO^*** animals, which lack expression of the *Rela* gene in neuro-ectodermal cell populations including neurons and macroglial cells but not in microglia, and normalized against the intrinsic telomerase activity of 1,000 HeLa cells. In contrast to the hypothesis of a reciprocal TERT:RelA induction, telomerase activity in these aged *Rela****^CNS-KO^*** mice was not altered relative to age-matched controls (Figure 7A; *P* = 0.326; *n* = 4 - 6). In accordance, no difference was observed between the RTL of cortical neural cells isolated from 19-20 months old *Rela****^CNS-KO^*** and age-matched control mice (*P =* 0.991, Figure 7B) when assessed by means of qPCR. Both data suggest that, in contrast to the discovery in tumor cell entities and blood cells [21,22], TERT:RelA might not establish a reciprocal interplay in the aging CNS.

## DISCUSSION

Senescence is defined as an irreversible cell cycle arrest provoked by telomere attrition in replication-competent cells devoid of sufficient telomerase activity. However, already in 1997 Coviello-McLaughlin and Prowse provided evidence that telomere dynamics in brain tissue might not follow this telomere hypothesis of replicative aging. They demonstrated that telomeres in brain of *M. spretus*, a mouse strain that harbors telomeres with a similar length as in humans, shorten unrelated to cell turnover and telomerase activity and even with stronger slope than in other tissues with restricted cell turnover. This was observed postnatally, whereas telomere shortening under aging was not detected [23]. Therefore, whether cell cycle cessation already at time of neuronal maturation predisposes to a senescent phenotype, or whether constitutive cell cycle exit, on the contrary, prevents from telomere shortening and thus retains a ‘juvenile’ state of the telomeric chromatin at higher ages, is not known. Accumulating evidence from more recent studies now indicates that penetrance of senescence characteristics is not restricted to proliferation-competent cells. Post-mitotic neurons of the CNS acquire a senescence-like phenotype as a result of dysfunctional telomeres and DNA damage [24]. Moreover, telomeres are a preferred site of persistent DDR under aging as well as stress conditions, irrespective of the presence or absence of telomerase activity [25,26]. Thus, telomere dysfunction plays a key role both in stress-induced premature senescence, which is generally independent of telomere shortening, as well as in DDR elicited by replication-mediated telomere attrition. However, telomere length integrity and dynamics in the working syncytium of the brain, which is exceptional for the lack of true cell division cycles of mature neurons, the limited cell turnover in glial populations and the capacity of differentiated neurons for unscheduled re-induction of abortive cell cycles [27], are still widely unexplored.

In our study, Flow-FISH-based telomere length analyses in highly aged mice, stratified for cell-cycle activity, reveal that telomere erosion indeed occurs in the aging rodent brain, and evidence that this telomere attrition is not restricted to neural populations with regular (e.g. in glia) or unscheduled (in distinct neurons) cell cycle activity, but also afflicts G_0_ phase-arrested neural populations devoid of any cell cycle activity, although to a lesser extent. Loss of RTL in the aged brain was further confirmed by a standard qPCR-based approach, irrespective of cell cycle activity. Additionally, by means of neuron-specific telomere length measurements, we directly identified neurons to contribute to the telomere shortening process. In conclusion, the brain appears to lose telomere length in both replicative and non-replicative cell moieties though at different rates, and thus shares aging features fundamental to many replicative organs.

To the best of our knowledge, this is the first study to evaluate telomere length dynamics in brain as a function of cell cycle activity. So far, the only work on aged murine CNS, which was performed in *M. spretus* displaying telomere lengths similar to humans, did not reveal telomere attrition when comparing 24-30 months old brain samples with 5 months old specimens, but described a decline in telomere length in the first postnatal months [23]. A similar reduction in brain telomere length during postnatal maturation was revealed in rat [28]. Apart from such time windows defining the phase of neuronal network consolidation [29], higher age categories are more frequently addressed in studies aiming to clarify the role of critically short telomeres in cellular senescence and life expectancy, as it is assumed from replicative tissue, and currently under analysis in rodents and humans [4,30–32]. Likewise, an age-related rise in the moiety of short telomeres (≤ 6 kb) was found in human centenarians in replicative tissues and corresponding cerebral cortices [4], whereas an age-associated alteration in the mean telomere length was not detected [3]. Thus, the impact of lifetime on mean telomere length in mammals is still understudied.

The few studies available on telomere length regulation in brain tissue support the notion that telomeres in the neural environment might not be static [3,4,32]. A direct comparison with rodent aging studies is hampered by multiple factors, as are the weak coverage of ages above 24 months, the heterogeneity of age categories and brain regions examined, the *per se* limited number of studies available on this topic and the variability of techniques applied to assess telomere repeats in interphase nuclei. Besides standard TRF length estimations based on Southern blotting, telomere length determination is often assessed by quantitative Q-FISH, or qPCR analyses. In addition, chromogenic *in situ* hybridization techniques, and more recent developments using the CRISPR/Cas9 technology and pyrrole-imidazole polyamides, may be suitable for further adaptation of telomere length determinations particularly in the CNS [33,34].

Here, we used a Flow-FISH-based approach, which has already been applied for RTL measurement in several cell types including blood cells [35] and keratinocytes [36], to bridge current knowledge on interphase and metaphase cell entities, and elucidate telomere length dynamics in the CNS environment, where replicative and quiescent cell populations are coexisting during the entire lifetime. While reduced self-renewal of stem cell pools in brain hippocampus has been associated with age-related learning and memory deficits [37], increased but atypical cell cycle activity in neurons is similarly assumed to sustain dysfunction of neurons and has been linked to apoptosis-like neuronal death [38,39]. In support of a related study by Fischer *et al.* [9], we provide evidence that cell cycle re-initiation is involved in the brain aging process, as indicated by the flow cytometry-based DNA content analysis. Deciphering cell entities with immanent scheduled or atypical cell cycle and their contribution to telomere attrition in the aging CNS is a topic of our current investigations. Also, the question whether neurons with aberrantly re-induced cell cycle activity are substantially contributing to the pool of neurons with shortened telomeres is a subject being currently under investigation by the authors. Apart, disturbances in telomere trimming processes [40] or induction of rapid telomere deletion, e.g., due to the loss of T-loops [41] may putatively also provide molecular bases to explain the observations described in this study.

Pursuing the hypothesis that both neurons and glia populations contribute to telomere length attrition in the aged brain, we further specified RTL measures for neuronal cell entities of our cortical isolates. Using an innovative NeuO- and FACS-based separation protocol to select vital neurons, and a qPCR-based telomere length measurement, we revealed remarkable age-related telomere curtailment in neurons already at the age of 24 months. These data show that neurons, though being a quiescent cell population with no replicative potential or with atypical cell cycle activity only, still undergo telomere attrition. Thus, neurons also account for the telomere length reduction observed for the G_0_/G_1_ population. The mechanism responsible for telomere attrition in non-dividing cells is still undefined and deserves further cytogenetic investigations. Telomeres are unable to perform non-homologous end joining in order to repair DNA double strand breaks [42] and thus are a site of persistent DDR, e.g., in response to oxidative or genotoxic stress [25,26]. Alongside, the number of DNA damage events within the telomere repeats including end caps strongly increases with age. This susceptibility of the telomere chromatin is obviously independent of both, absolute telomere length and presence of telomerase activity [25]. Therefore, since telomere attrition is not a prerequisite for continued DDR at telomeres [25], the inability to resolve DNA damage might itself predispose to a telomere shortening process and partially explain telomere instability in the brain with restricted replicative turnover. Likewise, in neurons with high energy consumption, age-related accumulation of reactive oxygen species (ROS) [13], as described for other cell entities [43], might damage guanine-rich sequences within the telomere structure [44,45]. Though ROS-mediated telomere shortening has so far been shown only for replicative tissue *in vitro*, it may also affect fragile telomeres in non-dividing neurons, or in neurons that re-induce atypical cell cycle activity. In support of this notion, telomere dysfunction and damage were causally linked to a senescence-like phenotype in replication-incompetent neurons [24]. Thus, in expansion of the work by Jurk and colleagues, who supported a process of cellular senescence also in post-replicative neurons [24], with features similar to those characterized for other cell types [26], we demonstrate here that telomere erosion is not restricted to replicative cell entities and thereby broaden our understanding of how the brain ages. Consistent with this finding, telomerase activity, which can also protect cells from oxidative stress and DNA damage, was not increased. Whether telomere shortening in neurons is an ultimate consequence of maturation-dependent loss in telomerase activity, a direct consequence of high ROS-mediated DDR, or a defective control, e.g., of telomere trimming strategies, is an interesting question, which has not been resolved yet.

Telomere tracts can be replenished by telomerase activity. TERT, the catalytic moiety of the telomerase enzyme, additionally exerts extra-telomeric functions, e.g., by mediating cell proliferation and apoptotic resistance in tumor cells [21], and by involvement in the defense against ROS through nucleus-to-mitochondria re-localization in response to oxidative stress in human fibroblasts [46]. Importantly, these non-canonical functions have been validated also in neurons with low levels of telomerase activity [47], that declines rapidly already during postnatal maturation of the growing CNS [48]. According to such beneficial roles, re-induction of telomerase activity in aged somatic cells, both of humans and mice, was capable to restore cellular replicative potential and correlated with improved tissue repair and features of rejuvenation, even in the largely postmitotic brain, and was also associated with a prolonged lifespan [49,50]. In contrast, as shown in lower organisms, early telomerase inactivation can accelerate aging independently of the telomere length [51]. Here, we observed telomerase activity levels of murine cerebral cortices to remain on a seemingly low, but stable level from young mature to high ages, at least when evaluated in whole cell lysates without a specific view on compartment-specific regulations. Future telomere length assessments in conditional TERT knockout animals will help to further define the role of TERT in the aged brain.

Apart from telomerase enzyme activity on chromosomal ends, alternative beneficial impact of TERT protein has been linked to healthy CNS aging [14] and improved survival of neurons [17]. Here, assessment of the TERT subunit in purified cellular sub-compartments of the murine neocortex revealed an aging-associated increase in TERT protein selectively in the cytoplasmic and membrane-bound fractions. Conversely, notable TERT protein levels in the chromatin-bound nuclear fraction, suggestive to represent telomere-associated telomerase function, was lacking both in the young as well as in the aged murine cortex, and thus is in line with the detected aging-mediated telomere attrition process. These findings are also in close agreement with a recent study reporting similar spatial TERT protein distributions in the murine neocortex, with highest TERT protein levels detectable in the cytoplasmic fraction and lowest levels found in the DNA-bound fraction [12]. Such compartment-specific TERT protein content paralleled an inverse regulation pattern for telomerase activity, when assessed at corresponding ages [12]. Notably, even replicative tumor cell lines were shown to lack relevant nuclear TERT protein, but to induce telomerase activity in the nuclear compartment by cytoplasmic-to-nuclear transfer of TERT protein only upon stimulation [52].

TERT protein re-localizations have been described for different cell types [14,52]. However, in our study, the low protein levels of TERT observed for either cellular compartment in the young brain strongly argue for a local induction of TERT protein synthesis by the aging process, rather than true protein relocation. Thus, whereas the low to absent levels of TERT protein in the soluble and chromatin-bound nuclear fractions might contribute to explain the aging-mediated telomere attrition detected, the biological role of TERT protein up-regulation or prevalence in alternative cellular compartments such as in the cytoplasm of neurons [17] and the membrane fraction, as detected here, still has to be defined. Notably, TERT content in the cytoplasmic sub-fraction increases under stress conditions [53]. Moreover, in cancer cells exposed to genotoxic stress, a low nuclear but high cytoplasmic/mitochondria content of TERT protein was correlated with lower levels of ROS, DNA damage and apoptosis [14].

Telomerase enzyme is regulated in a triplicate, i.e. on the transcriptional, protein and activity level via the transcription factor NF-κB involving the RelA/p65 subunit [52,54,55]. The reciprocal interaction comprises the induction of alternative telomerase functions, as shown in tumor cells [21,52], and the canonical regulation of telomere length [56,57]. Moreover, NF-κB impacts on both, TERT protein and enzyme activity, by influencing their cytoplasm-to-nuclear relocation [52]. Whether NF-κB regulates telomerase activity also in the aging brain, by binding to the TERT promoter as a downstream target and via a shift of the TERT protein and enzyme activity to the nuclear compartment has remained an open question. Therefore, we analyzed telomerase activity and RTL in the presence and absence of NF-κB *Rela* in the aged murine brain, and further compared TERT protein levels in different cellular compartments under young and aged naïve conditions. Though transcript levels of *Rela* in the aged murine cortex were significantly higher as compared with young tissue, this increase was not accompanied by a corresponding increase of telomerase activity, at least when assessed in whole cell preparations. Noteworthy, Akiyama and colleagues identified NF-κB-mediated nuclear induction of telomerase activity in replicative human MM.1S myeloma cells only when stimulated with TNF-α and when specified for cellular sub-fractions [52]. However, due to low levels of TERT protein under our experimental conditions, such a separation could not be performed for brain tissue. Whether the unchanged telomerase activity in *Rela****^CNS-KO^*** animals as compared to age-matched controls observed here might be due to a substitution of RelA functions by other subunits of canonical NF-κB, or by different molecular mechanisms, requires further analyses.

In summary, our data show that both, cell cycle-dependent and -independent changes in telomere length occur in the aging brain, which are assessable by application of current methodologies on interphase nuclei. Using a cell cycle phase-related approach, we reveal cell cycle activity to be a susceptibility factor, but not an obligatory criterion for telomere length reduction in neural cell populations. We thus assume additional factors such as, e.g., metabolic, inflammatory and genetic influences to be involved in telomere attrition in the CNS. Moreover, we provide direct evidence that non-replicative neurons contribute to the age-associated telomere attrition process found in the brain. The paralleled up-regulation of NF-κB *Rela* might mirror a direct link between its involvement in age-related genetic reprogramming and chromosomal aging. Thus, this study adds novel cellular and molecular aspects to a better understanding of the aging process of the CNS, where replicative senescence only partly explains cellular senescence.

## MATERIALS AND METHODS

### Ethics statement

Investigations were conducted in compliance with the ‘Animal Research: Reporting of In Vivo Experiments (ARRIVE)’ guidelines, executed in accordance with current European Union regulations and the Protection of Animals Act, and approved by the regional animal welfare authorities of Thuringia (accreditation number: 02-046/14).

### Animals

Male wild type mice of a C57BL/6 subcolony featuring hermetic inbreeding conditions were included at 3-4 and 25-27 months of age for RTL measurements, and up to 28 months for TRAP assay execution. In addition, 18-23 months old transgenic animals displaying a conditional, Cre-loxP-based nestin promoter-driven deletion of NF-κB *Rela* in neuro-ectodermal CNS populations, i.e. neurons, oligodendrocytes and astrocytes, but not microglia were used (further denoted as *Rela****^CNS-KO^***). Prove of *Rela* knockdown in these constructs has been carried out recently [58,59]. Age-matched littermates, in which exons of the *Rela* gene were flanked by *loxP* sequences but did not express Cre-recombinase served as controls (*Rela^Flox^*). C57BL/6 animals were housed in the humidity-, temperature-, and light/dark cycle-controlled animal facility of Jena University Hospital (Jena, Thuringia, Germany), with food and water available *ad libitum*. The *Rela****^CNS-KO^*** and *Rela^Flox^* strains were bred and accommodated accordingly at the Fritz-Lipmann Institute Jena.

Animals were deeply anesthetized with volatile isoflurane to achieve state of surgical tolerance, or killed by CO_2_ inhalation and it was the case for *Rela^CNS-KO^* and *Rela^Flox^* animals, prior to transcardial perfusion. Perfusion was performed with ice-cold PBS/Tris-buffered saline (TBS) (5 ml/min) in order to eliminate cerebral blood residues and avoid artifacts by contamination with nucleated blood cells. Brains were immediately extracted from euthanized animals, and weight-adjusted tissue sample volumes were taken for further experiments.

### Isolation of cerebral cortical cells

Calcium-free Hibernate® A medium (BrainBits, LLC, Springfield, IL, USA) was supplemented with 0.5 mM GlutaMAX™ (Invitrogen, Darmstadt, Germany), 132 mM d-trehalose (Sigma-Aldrich Chemie GmbH, Taufkirchen, Germany), and 310 IU/ml DNase I (Sigma-Aldrich Chemie GmbH). Collagenase IA (Sigma-Aldrich Chemie GmbH), papain (Cell Systems Biotechnologie Vertrieb GmbH, Troisdorf, Germany), and ovomucoid (Sigma-Aldrich Chemie GmbH) were diluted in Hibernate® A working solution to the concentrations specified below. Cerebral cortex, freshly isolated from young and aged mice, was transferred to 3 ml of collagenase IA (1 mg/ml) and incubated for 30 min at 37°C under gentle agitation. Meanwhile, papain was diluted and activated at 37°C for 30 min. Cortices were successively treated with 3 ml of activated papain at 2 mg/ml and 1 mg/ml under slow agitation for 15 min at 37°C. Tissue dissociation was mechanically reinforced by trituration using a fire-polished and smoothened Pasteur pipette with a tip size of 0.7-0.8 mm. Remaining tissue pieces were allowed to settle, and the supernatant was removed, transferred on ice, and blocked with 0.1% ovomucoid. Tissue pieces were stirred and triturated in the pre-used papain solution (2 mg/ml) for 5-10 min until tissue dissociation was complete. Tissue lysis was blocked with an equal volume of 0.1% ovomucoid, and the lysate was successively passed through 100 µm and 40 µm cell strainers and centrifuged at 500 × *g* for 5 min. The supernatant was removed and the pellet was resuspended in 1 ml of PBS containing 132 mM d-trehalose. The final neural cell suspension was incubated on ice for 15 min. After cell number assessment using a Neubauer counting chamber, cells were utilized for Flow-fluorescence *in-situ* hybridization (FISH), immunofluorescence for neural cell-type identification, or processed for neuron-specific cell sorting.

### Identification of neural cell types by immunofluorescence

Isolated cortical neural cells derived from 3 and 27-month-old mice were fixed with 2% paraformaldehyde for 20 min at room temperature (RT), washed twice in PBS and incubated with 10% normal donkey serum for 3 h at RT to block non-specific binding sites. Neural cell suspensions, including a physiological profile of CNS inherent cell entities, were processed with rabbit anti-ßIII-tubulin (1:250; RRID:AB_262133; Sigma-Aldrich Chemie GmbH) to select neurons, rabbit anti-CA-II (1:100; RRID:AB_2065996; Santa Cruz Biotechnology, Heidelberg, Germany) to identify oligodendrocytes, rabbit anti-Iba-1 (1:250; RRID:AB_839504; Wako Chemicals GmbH, Neuss, Germany) to discriminate microglia, and rabbit anti-S100ß (1:1,000; RRID:AB_2620024; Synaptic Systems GmbH, Göttingen, Germany) to label astrocytes. Primary antisera were prepared in 10% normal donkey serum containing 0.3% permeabilizing Triton X-100 and incubated overnight at 4°C under slow rotation. Cell isolates were then washed twice with PBS and incubated with Alexa Fluor® 488-conjugated donkey anti-rabbit Immunoglobulin G (H+L) secondary antibody, diluted to 1:250 (Invitrogen) in 10% normal donkey serum containing 0.3% Triton X-100, for 30 min at RT. After a further washing step, cells were incubated with 4',6-diamidino-2-phenylindole (DAPI) for 5 min at RT to stain nuclear DNA. Cells were rinsed twice with PBS, mounted on glass slides and evaluated by confocal laser scanning microscopy (LSM 710; Carl Zeiss AG, Jena, Germany). Photomicrographs were taken with a 63x oil-immersion objective using appropriate lasers (488 nm laser for Alexa Fluor® 488; 405 nm laser for DAPI).

Cell type-specific proportions were calculated from digital photomicrographs of freshly stained cell preparations taken with an AxioPlan 2 fluorescent microscope equipped with an AxioCam HRC camera and connected to AxioVision Rel. 4.8 software (Carl Zeiss AG). For each cellular marker, 20 images from each of three preparations were taken with a 40 x objective and assessed for both total DAPI-positive cell density and marker-specific cell moieties in a 360 x 275 µm display window. On average, an overall of 500 cells were analyzed to determine age-dependent neural proportions.

### Flow-Fluorescence *In-Situ* Hybridization-based telomere length determination

Cortical neural cells representing a physiological profile of resident populations were freshly isolated from mice aged 3 and 25-27 months. For RTL measurement by Flow-FISH, a telomere peptide nucleic acid (PNA)/fluorescein isothiocyanate (FITC)-based kit for flow cytometry (Dako Deutschland GmbH, Hamburg, Germany) was used according to the manufacturer’s instructions, with minor additions. The tetraploid human Leukemia 1301 cell line (Sigma-Aldrich Chemie GmbH) with a predicted telomeric repeat length of ~ 90 kbp served as an internal reference. The cell line was cultured and expanded according to the manufacturer’s instructions. For DNA denaturation, isolated primary cells and Leukemia 1301 cells were heated for 10 min at 82°C in hybridization solution, containing 70% formamide with or without the telomere PNA-FITC probe supplemented with 30 µl of DNase I (310 IU/ml). The samples were then incubated at RT in the dark overnight. The next day, reference and sample cells were washed twice and incubated in PBS or a kit-based propidium iodide (PI) solution for at least 3 h at RT, in order to assess background and sample-specific fluorescence intensity, respectively. Cells were filtered through a 30 µm cell strainer into fluorescence-activated cell sorting tubes, and run on a FACSCalibur™ flow cytometer (BD GmbH, Heidelberg, Germany), using the FL1 channel to detect PNA-FITC and the FL3 channel to detect PI. Settings were optimized for detector, amplifier and threshold values and re-used for all other experiments. Calibration and blank beads were run as internal references for the mean fluorescence intensity (MFI). Samples were analyzed in technical duplicates from 4-8 biological replicates per group.

To assess the relationship between MFI and the cell cycle phase-specific DNA content of each sample, PNA-FITC-H(FL1-Height)-related MFI values were normalized against sample-specific DNA content (PI-A; FL3-Area) in a cell cycle-dependent manner, and compared between young and aged groups according to the following formulas:

**MFI** (PNA-FITC-H),G_0_/G_1_ **/** **MFI** (PI-A),G_0_/G_1_ and **MFI** (PNA-FITC-H),G_2_/M **/** **MFI** (PI-A),G_2_/M

In order to separately validate the hybridization of PNA-FITC probes with target sequences in interphase chromatin of murine cortical neurons, the murine lymphoma cell line L5178-R (ATCC, CRL-1722; LGC Standards GmbH, Wesel, Germany) was used as a positive control to demonstrate telomere-PNA-FITC-related FISH signals under both, inter- and metaphase conditions, using DAPI as DNA marker (Figure S1).

### Neural and cell cycle-dependent cell-sorting strategies

Cell-sorting analyses were performed using the Flow and image Cytometry data analysis Software FCS Express 5 Plus (De Novo Software, Glendale, CA, USA) equipped with the Multi-Cycle add-on software tool. First, neural cell populations were separated according to cell size and granularity/complexity via the forward light scatter (FSC) and side light scatter (SSC), respectively (Figure S4A). Doublets were excluded by gating dot plots of PI-A (FL3-Area) versus PI-H (FL3-Height) according to the algorithm described by Wersto et al. (2001) [60] (Figure S4B). For PI-based cell attribution to different cell cycle phases, a histogram was plotted after combining an inclusion gate for cells and an exclusion gate for doublets (Figure S4C). For cells gated into either the G_0_/G_1_ or G_2_/M phases, MFI was calculated for PNA probe/FITC-stained and unstained samples, respectively (Figure S4D). RTL was calculated by subtracting the MFI of PNA probe/FITC-unstained samples from the MFI of PNA probe/FITC-stained samples (denoted as specific MFI).

### Sorting of neurons

In order to determine neuron-specific telomere length dynamics in cortical isolates, neurons were sorted on the basis of the NeuroFluor™ NeuO marker for living neurons (StemCell Technologies, Köln, Germany), followed by FACS separation. To ensure efficient sorting, cell isolates were subjected to a debris removal step (Miltenyi Biotec GmbH, Bergisch Gladbach, Germany). Briefly, neural cell isolates were centrifuged at 300 × *g* for 10 min, the pellet was resuspended in 3 ml of D-PBS supplemented with 2 ml of debris removal solution and overlaid with 4 ml of D-PBS. The gradient was centrifuged at 3,000 × *g* for 10 min, which resulted in different phases and a small pellet. The upper transparent phase and ring structure containing most of the debris were discarded. The pellet was resuspended in the lower transparent phase and the volume was filled up to 15 ml with D-PBS. Cells were again centrifuged at 1,000 × *g* for 10 min, recollected and incubated with NeuO dye in BrainPhys™ Neuronal Medium (StemCell Technologies, Köln, Germany) containing 132 mM d-trehalose and 310 IU/ml DNase at a concentration of 1 µM per 200,000 cells for 90 min at 37°C. As a negative control, cells were incubated with staining media omitting the NeuO dye. Cells were again centrifuged, and the pellet was resuspended in 500 µl of D-PBS. To remove cell clumps, the suspension was filtered through a 30 µM cell strainer directly into FACS tubes.

Cell suspensions were then run on a BD FACSAria Fusion flow cytometer (BD GmbH, Heidelberg, Germany). For sorting of the NeuO^+^ cell fraction, a yellow-green laser (561 nm) with a filter at 610 nm was applied. Neural cells were first selected on the basis of size (FSC) and granularity (SSC) and specified as neurons based on their NeuO staining properties (Figure S5). Doublets were excluded and only singlets were considered by plotting FSC-H versus FSC-A (Figure S5A). The NeuO^-^ population was determined via the negative control sample (Figure SF5B). The NeuO^+^ gated neurons (Figure S5C) were then sorted and collected in 5 ml polystyrene tubes (ThermoFisher Scientific, Darmstadt, Germany) containing 2 ml of Hibernate A-Ca medium. Tubes were further filled with medium up to a total volume of 5 ml and centrifuged. The cell fraction was resuspended in 0.5 ml PBS, transferred to 1.5 ml Eppendorf tubes and centrifuged. The supernatant was aspirated, and the final suspension was snap frozen in liquid nitrogen and stored at -80°C. DNA was extracted as described in Supplementary Data and further processed for RTL determination by qPCR.

### qPCR based telomere length determination

In order to further evaluate changes in RTL of cortical neural cells and verify results obtained by Flow-FISH, a qPCR-based method was added as described by O’Callaghan and Fenech (2011) [16], with some modifications. Briefly, telomeric genomic DNA was amplified using oligonucleotide primers against the telomere repeat sequence and normalized against the single copy gene 36B4. For each experimental group, genomic DNA samples from 4-6 biological replicates were run as technical duplicates for both, telomere repeats and the 36B4 gene utilizing a Rotor-Gene Q thermal cycler (QIAGEN, Hilden, Germany). The primer pairs selected were identical to those described by O’Callaghan and Fenech (2011) [16]. The cycling profiles and the primer sequences are shown in Supplementary Tables S1 and S2, respectively.

RTL was quantified in terms of the ratio of telomere repeat copy number “T” to single copy gene copy number “S” (T/S-ratio). The relative T/S-ratio was determined by the 2-∆∆Ct formula as described by Cawthon [61], which determines how each DNA sample varies in its T/S-ratio relative to the T/S-ratio of a reference sample.

### Determination of telomerase activity via a telomeric repeat amplification protocol assay

Telomerase activity was measured in brain cortices of young and aged C57BL/6 mice, and in *Rela****^CNS-KO^*** and *Rela^Flox^* mice using a telomeric repeat amplification protocol (TRAP)-based kit (TRAPeze®, S7710; Merck KGaA, Darmstadt, Germany) according to the manufacturer’s instructions. Brief description is given in the Supplementary Data. Data were assessed in technical duplicates from 5 animals per group and represented as absolute values in terms of amoles/µl, or referenced against the telomerase activity of 1,000 HeLa cells diluted from a positive control stock of 10,000 HeLa cells, which was provided with the kit. When assessed in the linear detection range of the kit, Ct values of the probe samples (30-31 cycles) corresponded to the Ct values obtained with an amount of 1,000 HeLa cells (30 cycles), thereby reaching the lower detection limit of the kit for both sample types. In terms of telomerase activity, cortical probe samples of young and aged animals equalized to approximately 68% and 62% of the low enzyme activity intrinsic to 1,000 HeLa cells, respectively (data not shown).

### DNA content analysis

Freshly isolated cortical neural cells, individually harvested from different young and aged animals, were fixed by the drop-wise addition of 1 ml of 70% cold ethanol under stirring in order to avoid precipitate formation, and incubated overnight at -20 °C. The subsequent day, cells were divided 1:1, with each portion being supplemented with 1 ml of PBS and centrifuged at 1500 × *g* for 5 min. The supernatant was discarded, and washing steps were repeated. Per sample, 500 µl of trout erythrocyte nuclei (TEN) (Biosure®, Grass valley CA, USA) suspended 1:4 in PBS were added and centrifuged at 1500 × *g* for 5 min. The supernatant was discarded, and the corresponding cell moieties were resuspended either in 250 µl of PBS or an equivalent volume of PI solution (Abcam, Cambridge, UK) and incubated at 37°C in a heating block for 30 min. Cells were finally filtered into FACS tubes and run on a FACSAria Fusion device.

### Western Blot analysis of subcellular protein fractions

Subcellular fractions from cortices of 3 months old mice were generated by using the Subcellular Protein Fractionation Kit for Tissues (Thermo Fisher Scientific) according to the manufacturer’s recommendations. Protein concentrations were determined via Bradford Assay using the QuickStart™ Bradford Dye Reagent (Bio-Rad, Hercules, CA, USA). To validate purity, 10 μg of each subcellular fraction were processed by a standard SDS-PAGE-based western blot protocol. After blocking of non-specific epitopes, the membrane was incubated with primary antibodies against GAPDH (1:8,000; RRID:AB_561053; Cell Signaling Technology, Danvers, MA, USA) and Histone H3 (1:10,000; RRID:AB_302613; Abcam, Cambridge, ENG, UK) overnight at 4°C. Afterwards, the membrane was washed in TBS supplemented with 0.1% Tween®20 prior to incubation with the appropriate HRP-conjugated secondary antibody (1:5,000; RRID:AB_631746; Santa Cruz Biotechnology, Dallas, TX, USA). Following washing, the bands were detected by chemiluminescence using Immobilon Western HRP Substrate solutions (Merck) and documented by the LAS3000 (Fujifilm, Düsseldorf, Germany) and the corresponding software.

### Assessment of compartment-specific TERT protein levels

For compartment-specific analysis of TERT protein, subcellular fractions from cortices of 3 and 27 months old mice were obtained as described above. Control samples were generated from whole cell lysates of 3 and 29 months old murine cortices as well as from small intestine, ovary and skeletal muscle of 3 months old mice. Briefly, the tissue was homogenized in lysis buffer (0.32 M sucrose, 4 mM Tris-HCl at pH 7.4, 1 mM EDTA, 0.25 mM Dithiothreitol; pH 7.2; 1:5 w/v) supplemented with cOmplete™ Mini Protease Inhibitor Cocktail (Sigma-Aldrich Chemie GmbH) as recommended by the manufacturer. Tissue homogenates were sonicated, incubated on ice for 10 min and centrifuged at 10,000 × *g* for 10 min at 4°C. The supernatant was taken as whole cell lysate. Protein concentrations were determined as mentioned above. To validate the specificity of the TERT antibody (RRID:AB_1218137; Novus Biologicals, Littleton, CO, USA) a conventional western blot approach was performed prior to the protein level assessment by the Simple Western™ method. For this approach, 25 µg of protein were loaded applying the TERT antibody (RRID:AB_1218137) in a dilution of 1:250. As a second step, protein levels were assessed by the automated Simple Western™ technique (Wes™; ProteinSimple, San Jose, CA, USA) according to the company’s standard protocol, using a 12-230 kDa Wes Separation Module joint to an anti-rabbit Detection Module for Wes™ (ProteinSimple). Briefly, subcellular protein fractions or whole cell lysates were diluted with Fluorescent 5x Master Mix provided in the kit and denatured immediately before application to the system at a final concentration of 0.4 µg/µl. The automated immunoassay, which is based on an advanced capillary electrophoresis and matrix-dependent protein separation, included incubation with primary antibody against TERT (1:25; RRID:AB_1218137) and β-Actin (1:150; RRID:AB_10002039; Novus Biologicals). For optimal primary antibody titration, a saturation curve for each of the antibodies was generated. HRP-conjugated secondary antibody and the enzyme’s substrate for the chemiluminescent detection were provided within the kit. The resulting electropherogram was adjusted for peak identity and analyzed using the Compass for Simple Western™ software (ProteinSimple).

### Analysis of NF-κB *Rela* expression by quantitative real-time qPCR

Brain cortices (65-105 mg per hemisphere) were micro-dissected from animals aged 3 and 25-28 months, and RNA was extracted using QIAzol® lysis reagent (Qiagen, Hilden, Germany) according to standard protocols. Profiling of the total yield of 28S:18S rRNA by capillary electrophoresis confirmed integrity of sample-specific mRNA. The RNA content ranged from 2,400-4,500 ng/µl, as assessed by spectrophotometry (NanoDrop 2000c; Peqlab Biotechnologie GmbH, Erlangen, Germany). Mean ratios of 2.02 ± 0.004 and 2.17 ± 0.015 for absorbance at 260 nm/280 nm and 260 nm/230 nm, respectively, reflected purity of the RNA and excluded contaminations with protein or phenol. The RNA was diluted to 100 ng/µl and reverse transcribed to cDNA using the RevertAid First Strand cDNA Synthesis Kit (Life Technologies, Darmstadt, Germany).

Quantitative real-time PCR (qPCR) was performed using a Brilliant II SYBR® Green QPCR Kit (Agilent Technologies Deutschland GmbH, Frankfurt, Germany). A total of 25 ng of cDNA per sample were used in a final reaction volume of 20 µl according to standard protocols. The product was amplified using the cycling profile and primers as mentioned in Supplementary Tables S3 and S4, respectively.

Relative changes in steady state transcript levels were assessed using ΔΔC_t_ values. Data were referenced against *Gapdh* as a stably expressed housekeeping gene, which was co-amplified, and presented as fold changes relative to young animals. Data analyses were performed using Rotor-Gene Q Series Software 2.3.1 (Qiagen) on 5 age-matched biological replicates assayed as technical duplicates.

### Statistics

Data subsets are presented as the mean ± standard error of the mean (SEM), except for those on relative transcript levels of NF-κB subunits, which are given as the geometric mean ± SEM. Pairwise inter-group comparisons were statistically evaluated using the two-tailed Student’s *t*-test or One- or Two-way analysis of variance (ANOVA), according to the parameters addressed. For pairwise multiple comparisons the Holm-Šídák and Tukey procedures were applied as *post-hoc* tests. For multiple, two-sided comparison of MFI values between different age- and cell cycle-categories, a log 10 transformation preceded Two-Way ANOVA. For DNA content-related MFI assessments, a twofold standard deviation (SD) was calculated. *P* < 0.05 was considered statistically significant.

## Supplementary Material

Supplementary Figures

Supplementary Tables

Supplementary Data
