# Supplementary material for: Cell cycle-dependent and -independent telomere shortening accompanies murine brain aging
Source: Aging (Albany NY). 2018 Nov 20;10(11):3397–420. doi: 10.18632/aging.101655 (PMC6286833; doi:10.18632/aging.101655)
Supplement: Supplementary Figures [file aging-10-101655-s001.pdf]

SUPPLEMENTARY FIGURES

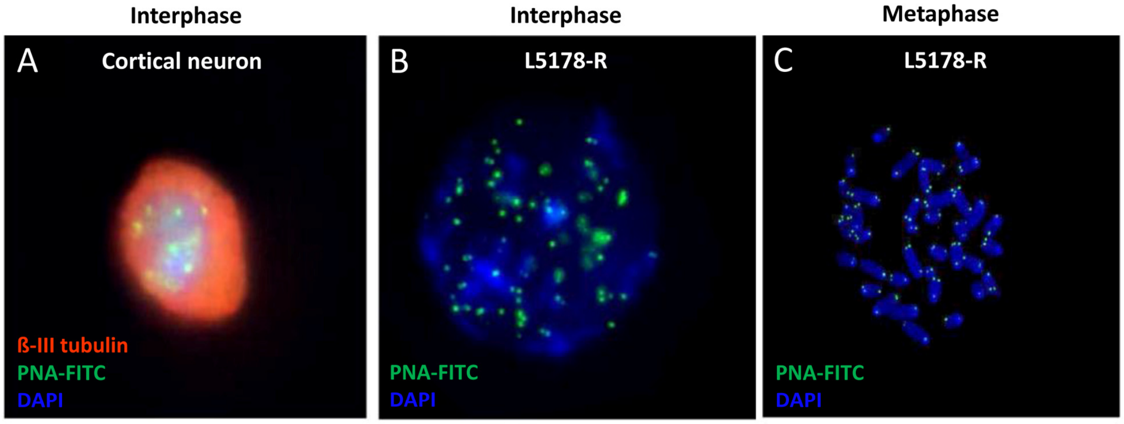

Supplementary Figure S1. Telomere-PNA-FITC-related FISH performed on murine interphase neurons and the murine lymphoma cell line L5178-R (LY-R). (A) Exemplified murine cortical neuron in interphase stained with the neuronal marker ß-III tubulin after FISH (red). (B, C) FISH performed on the murine lymphoma cell line LY-R in interphase (B) and metaphase (C). (A-C) Events of PNA-FITC probe hybridization with chromosomal TTAGGG sequence repeats are shown in green. DAPI was used as DNA marker (blue). 100 x objective applying oil immersion (METASYSTEMS ISIS).

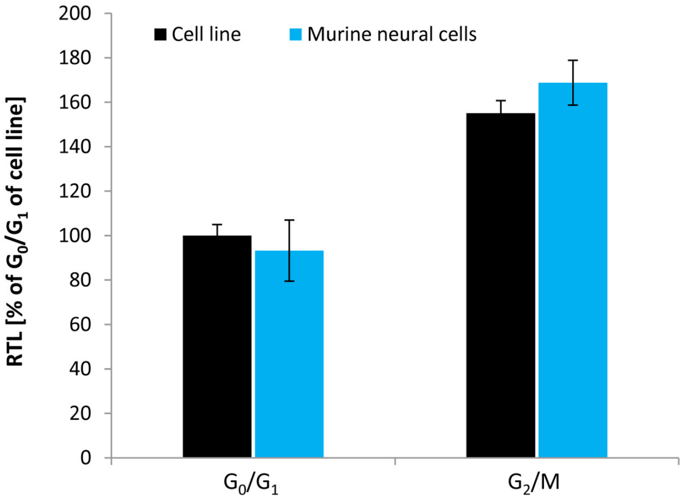

Supplementary Figure S2. Relative telomere length (RTL) of murine neural cells normalized against the human T-cell leukemia 1301 cell line. RTL of cortical neural cells isolated from 4.5-month-old inbred C57BL/6 mice was referenced against the 1301 cell line with a reported high telomere length of approximately 90 kbp. Analyses were separated for G_0/G_1 and G_2/M phases of the cell cycle (n = 3-4 for each condition). Cell cycle-specific RTL was normalized according to the following formula:

$$RTL = \frac{\text{(Specific MFI for sample cells) x DNA index of control cells x 100}}{\text{(Specific MFI for control cells) x DNA index of sample cells}}$$

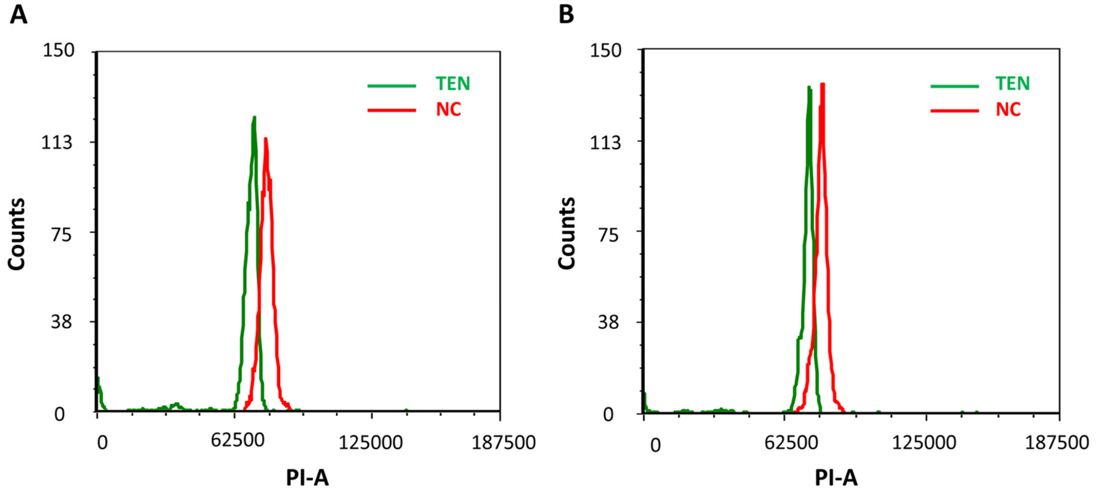

Supplementary Figure S3. **DNA content analysis.** (A, B) Representative DNA histograms assessed on the basis of PI staining of cortical neural cells (NC) isolated from young (A) and aged (B) murine brain. Trout erythrocyte nuclei (TEN) served as internal reference for DNA content assessment under both conditions. For each condition, *n* = 4. PI-A, propidium iodide area.

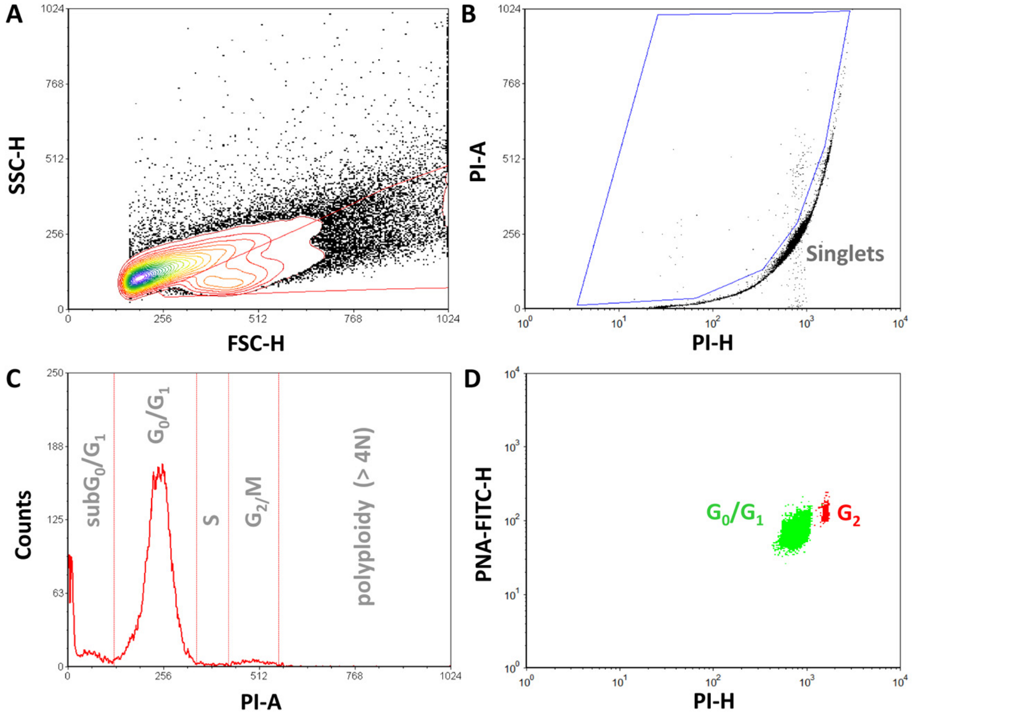

Supplementary Figure S4. **Gating strategies for RTL assessments**. Exemplified are cortical neural cells isolated from a 3-month-old B6 animal following FISH with PNA/FITC probes and DNA counterstaining with propidium iodide (PI). **(A) Cellular neural selection.** The contour plot is overlaid with the dot plot to better discriminate vital cells and debris. X-axis: forward light scatter, representing cell size. Y-axis: side scatter, reflecting cellular complexity and granularity. The red gate indicates vital neural cortical cells excluding other cell entities and debris. **(B) Exclusion of doublets.** The exclusion gate 'singlets' was applied by plotting PI-A (FL3-A) versus PI-H (FL3-H). The blue gate indicates the excluded doublet events. **(C) Cell gating according to cell cycle phases** using FCS Express 5 Plus plugin software. The histogram was plotted on the basis of DNA content following PI staining to gate the cells in different cell cycle phases. SubG_0/G_1 represents debris. The majority of neural cortical cells are found in G_0/G_1, with a minor cell population displaying features of G_2. Cells in S phase of the cell cycle, and with polyploid DNA content, are negligible under young conditions. **(D) Cell cycle-dependent PNA-FITC MFI.** Dot plot of PNA/FITC and PI stained neural cell moieties discriminated in (C), using PNA-FITC-H (FL1-H) and PI-H (FL3-H) as parameters to show the MFI for the cells in G_0/G_1 (green) and G_2/M phase (red) of the cell cycle.

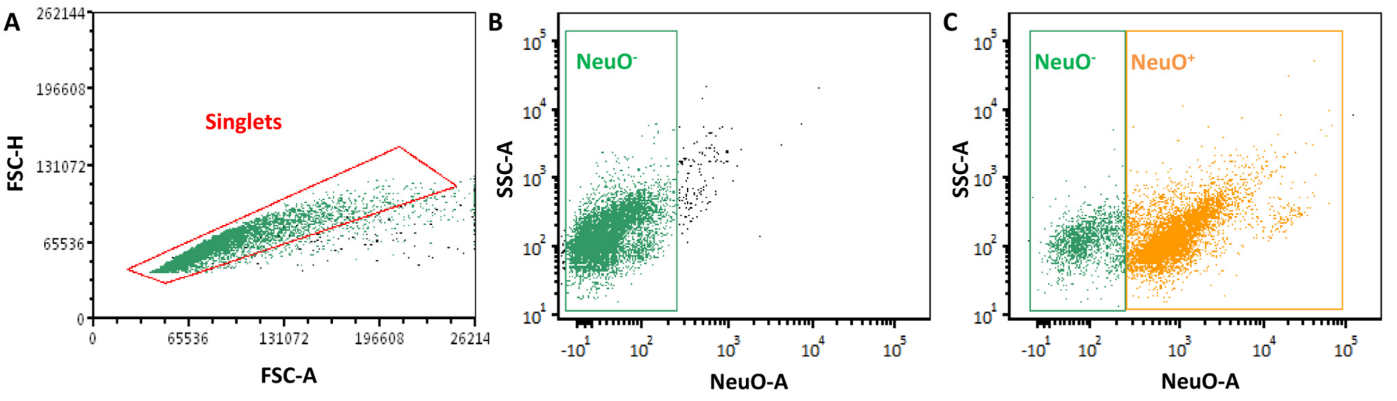

Supplementary Figure S5. C riteria for NeuO-based selection of neurons via FACS. Exemplified are dot plots of cortical neural cells isolated from young murine brain, illustrating the selection of singlets on the basis of FSC-A and FSC-H parameters (A), and the selection of NeuO-negative fractions ($NeuO^-$; B) and NeuO-positive cell moieties ($NeuO^+$; C) on the basis of NeuO-A and SSC-A. The NeuO-positive fraction indicates the neuronal population. For each condition, *n* = 4. SSC-A, side scatter area; NeuO-A, NeuO area; FSC-H, forward scatter height; FSC-A, forward scatter area.
