# Supplementary material for: Cell cycle-dependent and -independent telomere shortening accompanies murine brain aging
Source: Aging (Albany NY). 2018 Nov 20;10(11):3397–420. doi: 10.18632/aging.101655 (PMC6286833; doi:10.18632/aging.101655)
Supplement: Supplementary Tables [file aging-10-101655-s002.pdf]

SUPPLEMENTARY TABLES

Temperature °C	Time	Cycles
90	10 min	1
95	15 sec	40
60	60 sec	
Melting curve		

Supplementary Table S1. Thermal cycling profile used for RTL measurement in terms of T/S-ratios in brain. Depicted are the steps involved in amplification of the telomere repeat sequence and the 36B4 gene. To validate product specificity, a melting curve was generated at the end of each run. For further standardization, a “no-template-control” was included in all runs for both the telomere and 36B4 gene.

Gene	Primer sequence	Product size (bp)	Ref. PMID
TeloF	CGGTTTGTTTGGGTTTGGGTTTGGGTTTGGG TTTGGGTT	>76	21369534
TeloR	GGCTTGCCTTACCCTTACCCTTACCC TTACCCTTACCCT		
36B4F	ACTGGTCTAGGACCCGAGAAG	78	
36B4R	TCAATGGTGCCTCTGGAGATT		

Supplementary Table S2. Details on primers applied for RTL determination by qPCR. Given are the primer sequences and respective product sizes. Primers were designed to amplify genomic telomere DNA , which was normalized against the 36B4 single copy gene. All primers were used at a final concentration of 0.1 µM. Ref. PMID gives the primer source (PubMed-indexed for MEDLINE).

Step	Temp °C	Time	Cycles
**Initial denaturation**	95	10 min	1
**Denaturation**	95	15 sec	40
**Annealing**	60	30 sec	
**Elongation**	72	30 sec	

Supplementary Table S3. Thermal cycling profile used to define transcript levels of the NF-κB subunits *Rela* and *c-Rel*. To verify specificity of the amplified product, a melting curve was generated at the end of each run. For further standardization, a "no-template-control" was regularly included.

Gene	Forward primer	Reverse primer	Product size (bp)	Ref. PMID
Gapdh	CAACAGCAACTCCCACTCTTC	GGTCCAGGGTTTCTTACTCCTT	164	18606480
Rela	GACCCTGACCATGGACGATC	CGGCTGTTCGATGATCTCCA	138	-
c-Rel	GGAAGTGTCAGGGGAGGAGA	GGCTACTTGGCGGTGTACAT	91	-

Supplementary Table S4. Primer sequences for amplification of *Rela*, *c-Rel and Gapdh* mRNA, and product sizes. Primer sequences were designed to cover exon-exon boundaries in order to avoid genomic DNA amplification, and used at a concentration of 500 nM. *Gapdh* was amplified to normalize *Nf-κb* transcript levels. PMID gives the primer source (PubMed-indexed for MEDLINE).
