# Supplementary material for: Cell cycle-dependent and -independent telomere shortening accompanies murine brain aging
Source: Aging (Albany NY). 2018 Nov 20;10(11):3397–420. doi: 10.18632/aging.101655 (PMC6286833; doi:10.18632/aging.101655)
Supplement: Supplementary Data [file aging-10-101655-s003.pdf]

SUPPLEMENTARY DATA

Supplementary Protocol for DNA extraction from cortical neural cells

DNA from disseminated neural cells was extracted using the NucleoSpin Tissue kit (Masherey Nagel, Düren, Germany) according to the kit's manual, with slight modifications. A total of 200,000-300,000 cells were re-suspended in a final volume of 200 µl of buffer T1. Then, 25 µl of proteinase K solution and 200 µl of buffer B3 were added, and samples were incubated at 70°C for 3 hrs. Samples were supplemented with 210 µl of ethanol and vortexed vigorously. Nucleospin columns were placed in the collection tubes and loaded with samples, which were centrifuged at 11,000 × *g* for 1 min. The flow-through was discarded and the columns were placed back in the collection tubes. Extracted DNA was washed by adding 500 µl of buffer BW and centrifuged for 1 min at 11,000 × *g*. The flow-through was again discarded. The DNA was washed by adding 600 µl of buffer B5 to the column and centrifuged for 1 min at 11,000 × *g*. Once again, the flow-through was discarded. Empty columns were centrifuged to dry the silica membrane. Columns were transferred to new 1.5 ml tubes, and 50 µl of pre-warmed buffer BE were added to the nucleospin column to elute the genomic DNA. Samples were incubated at RT for 2 min and centrifuged at 11,000 × *g* for 2 min. Quantification and purity determination of DNA was performed by spectrophotometry using a Nanodrop 2000/2000C device (ThermoFisher Scientific, Darmstadt, Germany). A ratio of absorbance of ~1.8 or higher at 260 nm/280 nm indicated purity of DNA and reflected that samples were free of proteins or other contaminants. For telomere length measurement by qPCR, samples were diluted to a concentration of 4 ng/µl. The stocks and dilutions were stored at 4°C.

Supplementary Protocol for TRAP assay-based evaluation of telomerase activity in brain cortical tissue

3-[(3-cholamidopropyl)dimethylammonio]-1-propane-sulfonate (CHAPS) lysis buffer, supplemented with RNase inhibitor at a final concentration of 100-200 IU/ml, was added to frozen cortical tissue harvested from specified animal groups. Tissue was homogenized on ice using a motorized pestle. The volume of lysis buffer was adjusted to the wet weight of the cortical samples to achieve a final concentration of about 2 mg/µl (w/v). Following homogenization, the tissue lysate was incubated on ice for 30 min. Samples were centrifuged at 12,000 × *g* for 20 min at 4°C, and the supernatants were collected. Sample-specific protein content was measured via Bradford assay and adjusted to a final concentration of 500 ng/µl by dilution in CHAPS lysis buffer. For the polymerase chain reaction (PCR)-based TRAP assay, 1 µg of protein was used for each reaction.

To quantify telomerase activity based on the PCR amplification products, a standard curve was created from serial dilutions of a stock solution of the chemically synthesized TERT substrate TSR8 (telomeric substrate, 8 repeats, 20 amoles/µl), with final concentrations ranging between 0.02-2 amoles/µl of telomerase activity. To standardize the assay, all internal positive and negative controls were run in each PCR plate. Samples were incubated with type-specific (TS) primers provided in the master mix for 30 min at 30°C. To inactivate telomerase activity and amplify telomere repeats, a hot-start PCR protocol was used. Briefly, the mixture was heated to 95°C for 2 min. A total of 45 PCR cycles were proceeded as follows: 94°C for 15 sec; 59°C for 60 sec; and 45°C for 10 sec. Fluorescence energy transfer (ET) primers, which emit fluorescence signals only when incorporated into PCR amplification products, were employed to reduce amplification artifacts. Telomerase activity was assessed by measuring real-time fluorescence emission (in arbitrary units [a.u.]) corresponding to the average cycle threshold (Ct) value of each well and condition, and compared with the emission signal of the TSR8 product.
